# Impact of Fungal MAPK Pathway Targets on the Cell Wall

**DOI:** 10.3390/jof4030093

**Published:** 2018-08-09

**Authors:** Jacky Chow, Marysa Notaro, Aditi Prabhakar, Stephen J. Free, Paul J. Cullen

**Affiliations:** Department of Biological Sciences, SUNY-Buffalo, 341 Cooke Hall, Buffalo, NY 14260-1300, USA; jackycho@buffalo.edu (J.C.); marysanotaro@gmail.com (M.N.); aditipra@buffalo.edu (A.P.)

**Keywords:** cell wall, mannoproteins, cell–cell interactions, cell wall stress, MAPK

## Abstract

The fungal cell wall is an extracellular organelle that provides structure and protection to cells. The cell wall also influences the interactions of cells with each other and surfaces. The cell wall can be reorganized in response to changing environmental conditions and different types of stress. Signaling pathways control the remodeling of the cell wall through target proteins that are in many cases not well defined. The Mitogen Activated Protein Kinase pathway that controls filamentous growth in yeast (fMAPK) was required for normal growth in media containing the cell wall perturbing agent Calcofluor White (CFW). A mass spectrometry (MASS-SPEC) approach and analysis of expression profiling data identified cell wall proteins and modifying enzymes whose levels were influenced by the fMAPK pathway. These include Flo11p, Flo10p, Tip1p, Pry2p and the mannosyltransferase, Och1p. Cells lacking Flo11p or Och1p were sensitive to CFW. The identification of cell wall proteins controlled by a MAPK pathway may provide insights into how signaling pathways regulate the cell wall.

## 1. Introduction

The fungal cell wall is an external structure that is present in many fungal species. It provides a protective barrier that contributes to cellular properties including cell shape and adhesion [1,2]. Studies of the fungal cell wall come from model systems, like the budding yeast *Saccharomyces cerevisiae* [3,4,5], and from fungal pathogens, which, among other things, utilize the cell wall to evade the host’s immune system [6,7,8,9,10,11,12]. In pathogens, the fungal cell wall is important for infections and is a common target for anti-fungal drugs [8,9,13,14,15,16,17,18].

The cell wall is composed of proteins and polysaccharides [19]. Depending on the organism, the cell wall material can comprise 20 to 30 percent of the total biomass of the cell [20]. In species ranging from *Candida albicans* to *S. cerevisiae*, the major polysaccharides are β-1,3-glucan and β-1,6-glucan [4,5,10,21]. The cell wall is comprised of a three-dimensional lattice that is both a rigid structure that surrounds the cell and a malleable material that allows for growth and cellular expansion. In addition to testing for sensitivity to cell wall perturbing agents, new biosensors have been developed to measure the sensitivity of the cell wall [22].

The cell wall is composed of a diverse array of glycosylated proteins that can be cross-linked to the glucan network of polysaccharides [19]. Traditional approaches, and more recently genomics [23,24] and proteomics approaches [25,26], have identified cell wall proteins that connect to and regulate multiple cellular processes. Many of these proteins are glycosylated, which can influence their delivery to the cell exterior and their attachment to the cell wall [27,28,29,30]. Proteins can be cross-linked to the cell wall by a group of glycosylhydrolase/glycosyltransferase enzymes that modify oligosaccharides on glycosylated proteins [31] and by proteins that enzymatically attach oligosaccharides to the sugar backbone of the cell wall, like Dfg1p and Dcw1p [32]. These modifications are important for the incorporation of cell wall proteins into the wall [33]. Accordingly, cells lacking these enzymes release cell wall proteins into the medium. Studies of the *och1*Δ mutant, which is unable to elaborate the outer chain mannan, demonstrate that outer chain mannans play an important role in the incorporation of cell wall proteins into the cell wall [33].

Signal transduction pathways can regulate the cell wall. One pathway is the protein kinase C (PKC) pathway [34]. Another pathway controls filamentous growth and is commonly referred to as the filamentous growth Mitogen Activated Protein Kinase (or fMAPK) pathway [35]. The fMAPK pathway is sensitive to the glycosylation state of cells and can be induced in mutants in which the integrity of the cell wall has been compromised [36,37]. A third pathway is the high osmolarity glycerol response (HOG) pathway [38]. Signaling pathways that regulate the cell wall do not function in isolation [38,39]. For example, the fMAPK and PKC pathways can work together. In one study, cells with a hyperactive PKC pathway were found to upregulate the activity of the fMAPK pathway [37]. Similarly, overexpression of genes encoding PKC pathway sensors, *WSC2* and *MID2*, stimulate the fMAPK pathway [26]. Interestingly, *WSC2* is a transcriptional target of the fMAPK pathway [40], which may indicate that the fMAPK pathway can regulate the PKC pathway. Communication among pathways allows regulation of the cell wall in response to stress and during changes in cell type.

A critical function of the cell wall is to regulate adhesion. In yeast, cells adhere to each other and to surfaces by cell adhesion molecules. Flo11p is a glycosylphosphatidylinositol (GPI)-anchored mucin-like glycoprotein and the major cell adhesion molecule in yeast [41,42]. Although it has formerly been grouped with other members of the Flo family of proteins (Flo1p, Flo5p, Flo9p, Flo10p, and Flo11p) [43,44], Flo11p has a distinct amino acid sequence and structure [45,46]. Flo11p and other Flo proteins have hydrophobic properties. Such proteins also have amyloid-like regions that can participate in a type of cell adhesion known as catch bonding [47,48,49]. Flo11p can also be shed from cells, which can impact cell adherence [50]. Studying the extracellular material surrounding fungal cells can account for fungal responses, ranging from biofilm/mat formation [51,52] to filamentous/invasive growth, to host–fungal cell interactions. The cell wall may impact adhesion through other mechanisms as well. After cytokinesis, yeast (but not hyphal) cells fully separate. In fission yeast [53] and *S. cerevisiae* [54], hydrolyases such as glucanases and chitinase are secreted precisely after primary septum formation to promote cell wall separation [55,56]. This may be delayed during filamentous growth, resulting in decreased cell separation and increased shared cell wall material. Indeed, filamentous (Σ1278b) background strains [57,58] have ~30-fold lower levels of chitinase activity than other strains [59].

Here, we examine several aspects of cell wall regulation in *S. cerevisiae*. We found that different pathways are responsible for cell survival in response to specific stresses (37 °C and Calcofluor white (CFW)) that compromise the cell wall. The fMAPK was necessary for growth in media supplemented with CFW, and the HOG pathway was necessary for growth at high temperatures, as has been reported [60]. In addition, we identified proteins by proteomics studies and analysis of expression profiling data that participate in cell wall function and that may be potential targets of the fMAPK pathway. These include Flo11p, Flo10p, Och1p, Tip1p, and Pry2p. One of these, Och1p, is a cell-wall remodeling enzyme that is important for proper cell wall function and for virulence in fungal pathogens [61,62]. Therefore, targets of MAPK pathways that regulate the cell wall may contribute to cell wall remodeling to combat stress and promote cell-type transitions.

## 2. Materials and Methods

### 2.1. Microbiological Techniques

Yeast strains (Table 1) were manipulated using standard methods [63,64]. To make Yeast Extract Peptone Dextrose (YEPD) + CFW media, 100 mg/mL of filter-sterilized Calcofluor white fluorescent brightener (Sigma-Aldrich Life Science and Biochemicals, St. Louis, MO, USA) was added to YEPD media after autoclaving at a final concentration of 50 µg/mL. Plates were photographed using Evolution MP Color Camera (Media Cybernetics, Rockville MD, USA) and Q Capture software (version Pro 5.1, Surrey, BC, Canada). Saccharomyces Genome Database was a valuable resource for the study (https://www.yeastgenome.org).

### 2.2. Quantitative Polymerase Chain Reaction (qPCR) Analysis

Cells were concentrated (OD A600 = 20) and spotted in 10 µL aliquots onto YEP-Gal (2% agar) for 24 h. Cells were spotted in six colonies per plate equidistant to each other and the plate center. All six colonies were harvested for each trial, and three separate trials were compared for each strain. The entire colony surface was scraped into 500 µL of distilled water, harvested by centrifugation, washed, and stored at −80 °C. RNA was harvested by hot acid phenol chloroform extraction as described [40] Samples were further purified using Qiagen RNeasy Mini Kit (Cat. 74104, Hilden, Germany). RNA concentration and purity was measured using NanoDrop (NanoDrop 2000C, Waltham, MA, USA). RNA stability was determined by 1% agarose TBE gel electrophoresis. cDNA libraries from RNA samples were generated using iScript Reverse Transcriptase Supermix (BioRad, 1708840, Hercules, CA, USA). qPCR was performed using iTaq Universal SYBR Green Supermix (BioRad, 1725120) on BioRad CFX384 Real-Time System using the indicated primers (Table 2). Fold changes in expression were determined by calculating ΔΔCt using *ACT1* mRNA as the housekeeping gene for each sample. Experiments were performed from at least three biological replicates, and the average values are reported. Error bars represent the standard difference between experiments. *p*-values were determined by the Student’s *t*-test.

### 2.3. Fixation of Cells for SEM

For some experiments, cells were grown for 16 h in liquid media shaking at 225 rpm at 30 °C. Cell density was assessed by optical density (OD A_600_). Cells were collected by centrifugation (6000 *g* for 5 min), and washed in 0.1 M sodium phosphate buffer pH 7.4 and diluted to about 10^6^ cells, which were concentrated by syringe filtration by a 0.2 micron Whatman nucleopore polycarbonate filter paper with a 10 mL syringe (GE Whatman, catalog #889-78084, Maidstone, UK; BD Syringe, #309604, Franklin Lakes, NJ, USA). Cells were rinsed with one round of buffer by syringe, fixed with 1% glutaraldehyde for 15 min, and rinsed again. Cells were treated by a graded series of ethanol washes (30%, 50%, 70%, 85%, and 100%) by syringe to dehydrate the samples. The filter paper was removed from the holder, placed in a Petri dish and treated with hydroxymethyldiazane (HMDS). Samples were placed at 4 °C for 16 h and imaged the following day. All solutions were filter sterilized before use and stored in clean containers free of corrosion products from autoclaving or recycled use.

For other experiments, cells were diluted to an OD A_600_ of 0.2 in 0.1 M sodium phosphate buffer pH 7.4 and spotted onto sterile microsieves (BioDesign Inc., catalog #NC0928010, New York, NY, USA) on YEPD semisolid agar (2%) media. Plates were inverted and incubated for 16 h at 30 °C. Microsieves were removed from plates with sterile forceps and placed in a sterile Petri dish. Cells on microsieves were fixed with gluteraldehyde and dehydrated with ethanol and HMDS as described above, except that the syringe filter system was not used.

### 2.4. Microscopy

For scanning electron microscopy, the FE-SEM (field emission scanning electron microscope) Hitachi SU70 (Chiyoda, Tokyo, Japan) was used to obtain electron micrographs in this study. For differential interference contrast (DIC) and fluorescence microscopy, a Zeiss Axioplan 2 microscope (Oberkochen, Germany) was used. Digital images were obtained with the Axiocam MRm camera (Zeiss, Oberkochen, Germany). Axiovision 4.4 software (Zeiss, Oberkochen, Germany) was used for image acquisition and analysis. Micrographs were taken at 100× magnification.

### 2.5. Quantitation of Colony Ruffling

Colonies were grown for two days on YEPD agar media and photographed. Images were imported into ImageJ (https://imagej.nih.gov/ij/). Radii were drawn from the center to the outer edge. A ruffle was identified by a light band with dark bands on either side.

### 2.6. Protein Preparation for MASS-SPEC Analysis

The proteomic approach involved the purification of cell walls, extraction of cross-linked proteins, separation of proteins by SDS-PAGE gel electrophoresis, and analysis by mass spectrometry (MASS SPEC). Comparisons of the proteome were made between wild-type strains and two mutants that have altered fMAPK activity. One mutant lacks a transcription factor that controls target gene expression (*ste12*Δ (PC1079) [71]). Another mutant lacks a transcriptional repressor that is expected to show elevated levels of fMAPK pathway target proteins *dig1*Δ (PC3039) [72,73,74]. Specifically, wild-type (PC538), *ste12*Δ (PC1079), and *dig1*Δ (PC3039) cells were grown in 250 mL of YEP-GAL liquid medium to mid-log phase. Cell walls were enriched in the following way: cells were transferred to ice-cold Fast-Prep tubes and subjected to 36 cycles of shaking on a Fast-Prep machine (MP Biomedical, Solon, OH, USA). Each cycle was for 20 s at speed setting 6 on the machine, and the samples were cooled on ice for 60 s between each cycle. Broken cell walls were collected by centrifugation (5000 *g* for 10 min) and washed twice in PBS. SDS (1%) was added to the resuspended cell wall preparation, and samples were boiled for 10 min to remove non-specific proteins. SDS-treated preparations were washed twice in PBS and treated for 30 min at 37 °C with 1% β-mercaptoethanol. Cell walls were collected and washed twice by centrifugation with ice-cold water to give purified cell wall preparations, which were lyophilized.

Cell walls were subjected to tri-fluoromethanolsulfate treatment to remove oligosaccharides as described [26]. Trifluoromethanesulfonic acid (TFMS) cleaves glycosidic bonds and leaves peptide bonds intact. The only sugars remaining on the protein are the *N*-acetylglucosamine residues from N-linked oligosaccharides, which are attached to asparginine sites. *N*-acetylglucosamine-asparginine was included as a possible amino acid in analysis to identify sites of N-linked oligosaccharide addition. Samples of proteins released from the cell walls by TFMS treatment were precipitated by the addition of trichloroacetic acid (TCA) and subjected to a short electrophoresis run so that the proteins entered a 4–12% polyacrylamide gel. The gel was stained with Commassie blue and the top part of the gel, containing the proteins, was cut from the gel with a razor blade and placed in a microfuge tube. Gel slices from a blank region of gel were included as a control.

### 2.7. Mass Spectrometry

Samples were analyzed by MASS SPEC analysis at the Fred Hutchinson Cancer Research Center. Gel pieces were cut from a Coomassie stained gel and subjected to tryptic digestion [75]. Tryptic peptides were concentrated and desalted using a C18micro ZipTip (Millipore, Burlington, MA, USA) following the manufacturer’s instructions. The eluted peptides were dried by vacuum centrifugation, resuspended in 20 µL of 2% acetonitrile/0.1% formic acid and 18 µL was analyzed by liquid chromatography coupled to tandem mass spectrometry (LC-MS/MS) with an Easy nlC-1000 (ThermoScientific, Waltham, MA, USA) coupled to an OrbiTrap Elite (ThermoScientific, Waltham, MA, USA) mass spectrometer using an instrument configuration [76]. In-line de-salting was accomplished using a reversed-phase trap column (100 µm × 20 mm) packed with Magic C_18_AQ (5-μm 200Å resin; Michrom Bioresources, Auburn, CA, USA) followed by peptide separations on a reversed-phase column (75 μm × 250 mm) packed with Magic C_18_AQ (5-μm 100Å resin; Michrom Bioresources, Auburn, CA, USA) directly mounted on the electrospray ion source. Chromatographic separations were conducted at a flow rate of 300 nL/min with an elution profile from 3% B to 7% B in 2 min, 7% B to 33% B in 90 min, 33% B to 50% B in 7 min, 50% B to 50% B in 3 min, and 50% B to 90% B in 5 min using 0.1% formic acid in water (A) and 0.1% formic acid in acetonitrile (B) as solvents. Data were collected in a data-dependent mode in which a high mass resolution/high mass accuracy scan (in the FT part of the instrument) was followed by low resolution/low mass accuracy MS/MS scans of the 15 most abundant ions from the preceding MS scan (in the LTQ part of the instrument). The FT part of the instrument was set at a target resolution of 120,000 at *m*/*z* 400, an automatic gain control (AGC) target value of 1e6, and a maximum ion time of 100 ms while the ion trap was set to a MSn AGC target value of 1e4 and a MSn maximum ion time of 100 ms. Normalized collision energy of 35% and isolation widths of 2.0 were used for MS2 events. Dynamic exclusion was enabled with a repeat count of 1, a repeat duration of 15 s, an exclusion duration of 30 s, a low exclusion mass width of 10 ppm and a high exclusion mass width of 10 ppm. Unassigned, +1, and +4 charge states were rejected.

Raw MS/MS data were analyzed by Proteome Discoverer v1.4 using the Saccharomyces Genome Database (www.yeastgenome.org) protein database (downloaded 111814) that was appended with protein sequences from the common repository of adventitious proteins (cRPA; www.thegpm.org/crap/). Met +15.995 (oxidation), Cys +57.021 (carbamidomethyl), and Asn +203.079 (hexNAc) were set as variable modifications. The mass tolerances were set to 10 ppm and 0.6 Da for precursor and fragment ions, respectively. The enzyme was set to Trypsin, and up to two missed cleavages were permitted. Peptide validation was conducted with Percolator and peptide identification results were filtered with false discovery rate of less than 1%.

## 3. Results

### 3.1. fMAPK Pathway Is Required for Viability in Response to Cell Wall Stress

**Figure 1:**
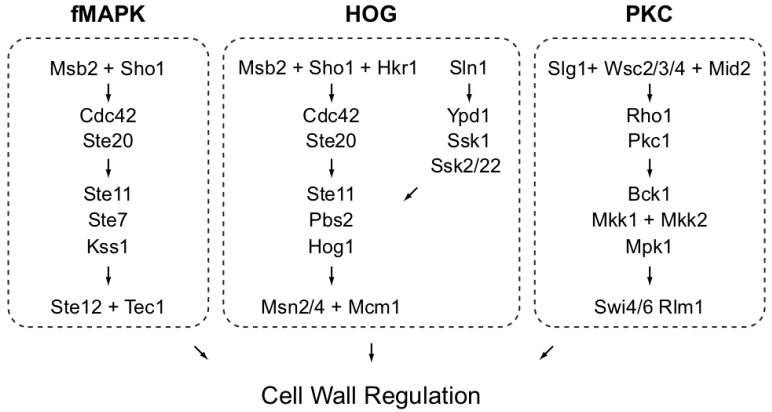
MAPK pathways that can regulate the cell wall in yeast. Not all proteins are shown, adapted from [77].

The aim of the study is to define the role of MAPK pathways in regulating the cell wall in yeast. At least three MAP kinase pathways regulate aspects of the cell wall (Figure 1).

**Figure 2:**
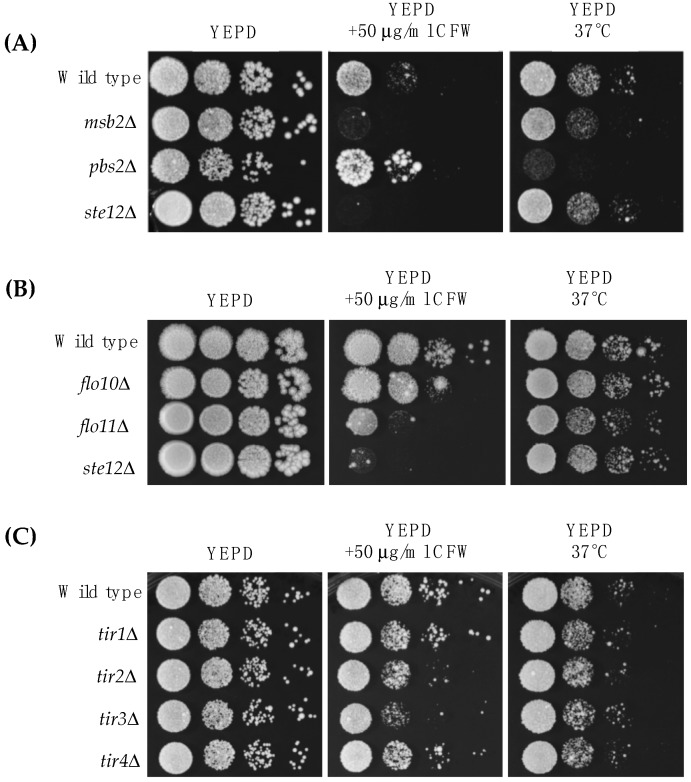
Role of MAPK pathways in regulating cell wall stress in yeast. (**A**–**C**) 0.1 O.D_600_ of wild type (PC538) and indicated strains (see Table 1 for strain numbers) were spotted in 10-fold serial dilutions onto YEPD media supplemented with or without CFW at a final concentration of 50 µg/mL. Plates were incubated at 30 °C or 37 °C and photographed after two days of growth (panels A and C) or three days (panel B).

Here, we focus on the MAP kinase pathway that controls filamentous growth (fMAPK pathway). We first tested whether the fMAPK pathway is important for survival in response to cell wall stress. Two fMAPK pathway mutants, *msb2*Δ (PC948) and *ste12*Δ (PC1079), showed a growth defect in media supplemented with Calcofluor white (CFW), a cell wall perturbing agent that binds to chitin [78,79] (Figure 2A, YEPD +50 μg/mL CFW). Msb2p, an established regulator of the fMAPK pathway [67], can also regulate the HOG pathway [80,81], so the role of the HOG pathway in the response to cell wall stress was examined. As previously reported [82], a HOG pathway mutant (*pbs2*Δ mutant (PC2053)) did not have a growth defect in CFW and in fact grew slightly better than wild-type cells. Growth at high temperatures, like 37 °C, can also compromise the cell wall [83]. The *pbs2*Δ mutant showed a growth defect at 37 °C (Figure 2A, YEPD 37 °C), in line with a previous study [60]. The fMAPK pathway was not required for growth at 37 °C (Figure 2A, YEPD 37 °C), which distinguishes it from the role that Msb2p plays in thermotolerance in *C. albicans* [84]. Therefore, the fMAPK and HOG pathways play different roles in cell wall integrity in response to two stresses.

### 3.2. Proteomic Analysis of the Yeast Cell Wall Identifies Targets of the fMAPK Pathway

We next sought to identify cell wall proteins that may be regulated by the fMAPK pathway. We previously utilized an approach to identify cell wall proteins in yeast [26]. Here, the approach was modified to enrich for fMAPK-dependent cell wall proteins. Cell-wall proteins were isolated from a wild-type Σ1278b strain of *S. cerevisiae* (PC538) and two mutants. One lacked an intact fMAPK pathway, (PC1079, *ste12*Δ [71]). The other showed elevated fMAPK pathway activity due to loss of a transcriptional repressor (PC3039, *dig1*Δ [72,73,74]). Cell wall extracts were prepared from the three strains and run on an SDS-PAGE gel. Total proteins were extracted from the gels and subjected to MASS SPEC analysis. Proteins absent in the *ste12*Δ mutant, or found exclusively in the *dig1*Δ mutant, were identified (Table 3). These proteins represent potential direct or indirect targets of the fMAPK pathway. Direct targets are defined as genes whose expression is controlled by binding by the transcription factors Ste12p and Tec1p to their cognate promoters [85]. As is typical for this type of MASS SPEC approach [26], some non-cell wall proteins were also identified but were not considered in the analysis.

**Figure 3:**
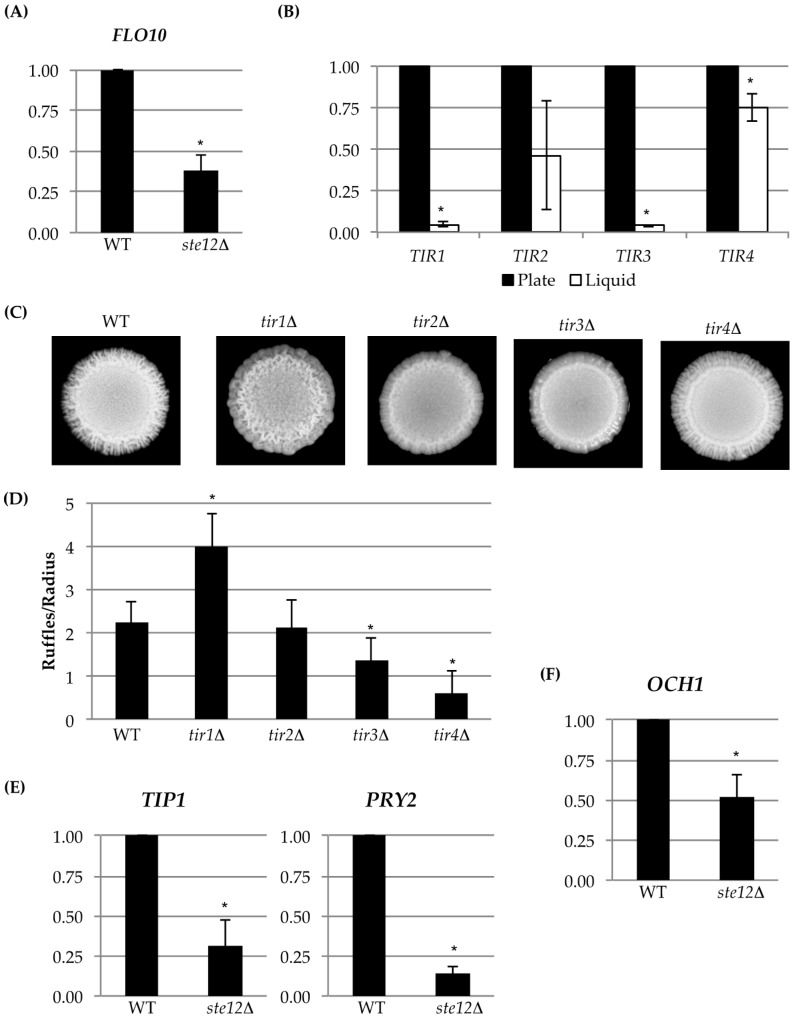
Analysis of cell-wall gene expression and mutant phenotypes. qPCR analysis was used to measure differences in expression levels of targets of identified by MASS SPEC analysis and gene expression profiling. (**A**) *FLO10*; (**B**) *TIR* gene family expression analysis from cells grown on agar media versus liquid. All asterisks are *p* < 0.05 for each pair; (**C**) colonies of wild-type (WT, PC538) cells and the indicated deletion mutants of members of the *TIR* family; (**D**) bar graph showing number of ruffles per radii in the wild type and the indicated *TIR* deletion mutants (strain numbers of *TIR* deletion mutants can be found in Table 1). Asterisks are *p* < 0.05 relative to WT. Additional qPCR analysis of targets identified in a comparative RNAseq experiment (Chow et al., under review; GSE115657) (**E**) *TIP1* and *PRY2*; (**F**) *OCH1* (WT, PC538; *ste12*Δ, PC1079). All asterisks are *p* < 0.05 relative to wild type.

Fourteen cell wall proteins were identified as fMAPK targets by MASS SPEC analysis (Table 3; see Tables S1–S4 for the complete experiment). Forty-six percent have been identified by a previous MASS SPEC approach [26], and all of the proteins are known to be in the cell wall (Table 3). The MASS SPEC data identified known transcriptional targets of the fMAPK pathway, including Svs1p [77] and Flo11p [41,42,86,87]. The MASS SPEC approach also identified two mating pathway cell wall targets Bar1p [88,89] and Fig2p [90], which are known to be regulated by pheromone response MAPK pathway that shares the same transcription factor Ste12p [88,91]. Another member of the Flo family of proteins, Flo10p [43], was also identified. We verified that Flo10p was a transcriptional target of the fMAPK pathway by quantitative real-time PCR (qPCR) analysis (Figure 3A). We also tested mutants lacking the Flo11p and Flo10p proteins for sensitivity to CFW and 37 °C. Interestingly, Flo11p was required for normal growth in media supplemented with CFW (Figure 2B; PC1029, *flo11*Δ). It may be that this abundant cell adhesion molecule also has a function in the organization or stability of the cell wall. Flo10p played a minor role in CFW sensitivity (Figure 2B; PC2912, *flo10*Δ). Its minor role may be consistent with the fact that most members of the Flo family are poorly expressed [43,44].

Another cell wall mannoprotein, Tir4p, was also identified by MASS SPEC analysis (Table 3). Tir4p is a member of a family of cell wall proteins (Tir1p, Tir2p, Tir3p, and Tir4p) [92,93]. *TIR* deletions were examined in CFW and 37 °C. Several *TIR* deletion mutants showed some sensitivity to CFW (Figure 2C) but not growth at 37 °C (Figure 2C). Though the regulation of Tir4p by fMAPK could not be confirmed by qPCR analysis, the entire *TIR* family of cell wall mannoproteins was highly expressed during growth on semi-solid agar as compared to growth of cells in liquid culture (Figure 3B), which is a condition that promotes biofilm/mat formation [52]. Thus, although the *TIR* genes may not be transcriptional targets of the fMAPK pathway, they are potential candidates of biofilm/mat growth. The four *TIR* encoded proteins regulated colony morphology, although each *TIR* deletion mutant had a distinct phenotype, such as differences in ruffling pattern and number of surface ruffles. Specifically, colony ruffling occurs due to adhesion among cells, and is a common feature of biofilm/mat growth [52,94]. The *TIR* deletion mutants showed different ruffled patterns (Figure 3C), which were quantitated by ImageJ analysis (Figure 3D). Thus, each *TIR* encoded protein may play a unique role in the development of colony morphology.

### 3.3. Analysis of Expression Profiling Data Identifies Och1p as an fMAPK Target That Regulates Cell Wall Ultrastructure

Comparative expression profiling can also identify transcriptional targets of signaling pathways. Analysis of comparative expression profiling data (Chow et al. under review, [40]), showed that a subset of the cell wall proteins identified by MASS SPEC analysis were differentially regulated by the fMAPK pathway (Table 3, *dig1*Δ and *ste12*Δ columns). Analysis of the expression profiling data identified three genes, the mannosyltransferase *OCH1* [31,108], *PRY2*, which encodes a pathogen related cell wall protein [107], and *TIP1*, which encodes a cell wall mannoprotein [106,109], as potential targets of the fMAPK pathway (Table 3). These genes were confirmed to be targets of the fMAPK pathway by qPCR analysis (Figure 3E,F). Pry2p is also involved in a sterol quality-control pathway and may be upregulated by the fMAPK pathway for this reason [107].

**Figure 4:**
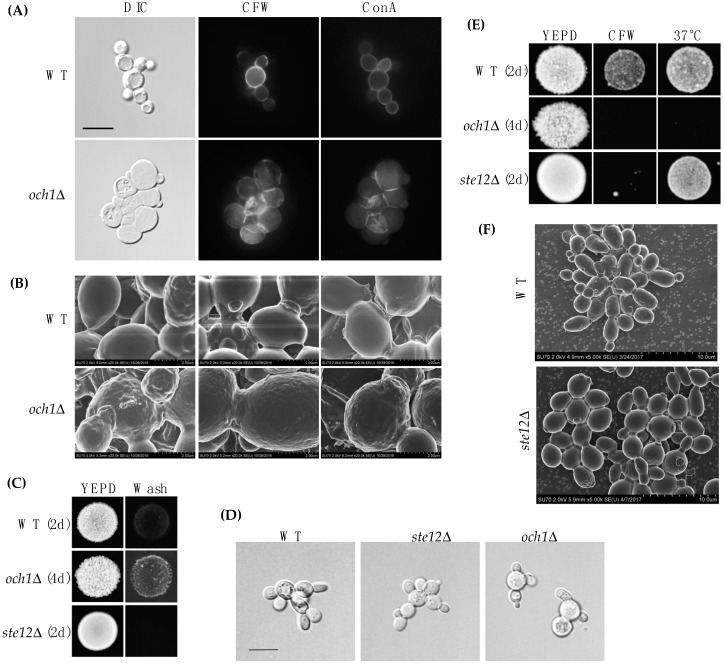
Analysis of the role of Och1p in cell wall organization. (**A**) wild type (PC513) and the *och1*Δ (PC514) mutant were stained with CFW (middle panel) and ConA (right panel), 100× magnification, scale bar 5 microns; (**B**) SEM of wild-type or *och1*Δ mutant showing different cell wall architecture, 20.0k× magnification; (**C**) plate-washing assay and of wild type (PC538), *ste12*Δ (PC1079) and *och1*Δ (PC7133). WT colony diameter, 0.75 cm; (**D**) single cell invasive growth assay, 100× magnification, scale bar 5 microns (**E**) cell wall sensitivity of the *och1*Δ mutant, WT colony diameter, 0.75 cm; (**F**) SEM of wild-type (PC538) cells and the *ste12*Δ (PC1079) mutant, 5.0k× magnification. Assays with the *och1*Δ mutant were shown at different days to account for the growth defect of the mutant.

Och1p was not identified by MASS SPEC analysis perhaps because it resides in the Golgi apparatus. *OCH1* may not be a direct target of the fMAPK pathway because Ste12p and Tec1p have not been identified at the *OCH1* promoter [96,110,111]. Another transcription factor that controls *OCH1* expression (e.g., Gcn5p [112], Med2p [112], Sfp1p [113], Skn7p [96,114], Spn1p [112], Spt6p [112], Swi4p [96], and Tup1p [114]) may be regulated by the fMAPK pathway, although we have not tested this possibility. The phenotype of the *och1*Δ mutant was examined in more detail. By DIC microscopy, *och1*Δ cells (PC514) were rounder and larger than wild-type (PC513) cells (Figure 4A). By fluorescence microscopy, the *och1*Δ mutant showed a defect in cell wall morphology by CFW staining (Figure 4A). Although it is not clear why the *och1*Δ mutant is sensitive to CFW, a chitin-targeted cell wall perturbing agent, the *och1*Δ mutant is sensitive to many chemical treatments, including caspofungin [115], which targets (1→3)-β-d-glucan synthesis in the cell wall [116]. By concanavalin A staining (ConA), the *och1*Δ mutant looked relatively normal (Figure 4A). The *och1*Δ mutant was also examined by scanning electron microscopy (SEM). Wild-type cells exhibited a relatively smooth cell wall, while the *och1*Δ mutant showed a wrinkled cell wall (Figure 4B).

Because of these differences, the *OCH1* gene was disrupted in the Σ1278b strain background (PC538). The *och1*Δ mutant (PC7133) did not have a defect in invasive growth compared to wild type cells (PC538) by the plate-washing assay (Figure 4C). However, by the single-cell invasive growth assay, the *och1*Δ mutant had morphological defects (Figure 4D). As expected, the *och1*Δ mutant had defects in growth in CFW and 37 °C (Figure 4E). These results validate previous findings that demonstrate the importance of the Och1p protein in cell wall integrity and ultrastructure [62]. Cells lacking an intact fMAPK pathway had relatively normal cell wall morphology (Figure 4F). Therefore, the mere reduction in *OCH1* levels in fMAPK pathway mutants does not result in a visible defect in cell wall ultrastructure but may nevertheless be important for cell wall remodeling by the fMAPK pathway.

## 4. Discussion

In the study, the role of signaling pathways (fMAPK and HOG) in regulating and maintaining the cell wall was examined. Different pathways were responsible for cell survival in response to different stresses. The fMAPK was necessary for growth in CFW, and the HOG pathway was necessary for growth at high temperatures [60].

We also identified and confirmed cell wall proteins as targets of the fMAPK pathway. These include Flo11p, Flo10p, Och1p, Tip1p, and Pry2p. By exploring the role of Och1p, we found that the protein is required for proper cell wall formation and morphology. The fact that Och1p is upregulated by the fMAPK pathway makes sense from the perspective that, during filamentous growth, cells spend more time in the apical phase of growth [117], where more cell-wall production may be needed at cell tips. In addition, Flo11p and Och1p were required for CFW resistance, which might account for the CFW sensitivity of fMAPK pathway mutants. Given that related adhesion and cell wall proteins also function in pathogens like *C. albicans* [118], the study may provide general insights into how MAPK-dependent regulation of the cell wall occurs.

## 5. Conclusions

Fungal cell walls can be reorganized by signaling pathways, which regulate the levels of target proteins. Here, we identify target proteins of a MAPK pathway that function in the regulation of the cell wall. Insights gained from the study may impact the understanding of how signaling pathways regulate the cell wall in other fungal species, including fungal pathogens.

## Figures and Tables

**Table 1:** *Saccharomyces cerevisiae* strains used in the study.

Strain (a)	Description	Reference
PJ69-4A (b)	*MAT*a *trpl-901 leu2-3,112 ura3-52 his3-200 ga14*Δ *ga18O*Δ *LYS2::GALl-HIS3 GAL2-ADE2 met2::GAL7-lacZ*	[65]
PC312	*MAT*α *ura3-52*	[66]
PC313	*MAT*a *ura3-52*	[66]
PC344	*MAT*a/MATα *ura3-52*/ura3-52	[66]
PC513 (c)	JHY7	J. Horecka
PC514 (c)	JHY7 *och1*	J. Horecka
PC538	*MAT*a *ura3-52 ste4 FUS1-lacZ FUS1-HIS3*	[67]
PC1029	*MATa ura3-52 ste4 FUS1-lacZ FUS1-HIS3 flo11::KanMX6*	[50]
PC948	*MAT*a *ura3-52 ste4 FUS1-lacZ FUS1-HIS3 msb2::KanMX6*	[67]
PC1079	*MAT*a *ura3-52 ste4 FUS1-lacZ FUS1-HIS3 ste12::URA3*	[67]
PC2053	*MAT*a *ura3-52 ste4 FUS1-lacZ FUS1-HIS3 pbs2::KanMX6*	[68]
PC2912	*MAT*a *ura3-52 ste4 FUS1-lacZ FUS1-HIS3 flo10::URA3*	[26]
PC3039	*MAT*a *ura3-52 ste4 FUS1-lacZ FUS1-HIS3 MSB2-HA dig1::NAT*	[68]
PC7133	*MAT*a *ura3-52 ste4 FUS1-lacZ FUS1-HIS3 och1::URA3*	This study
CB8E11 (d)	*MAT*a *can1Δ::Ste2pr-spHIS5 lyp1Δ::Ste3pr-LEU2 his3::hisG leu2Δ0 ura3Δ0 tir1Δ*	[69]
CB17H7 (d)	*MAT*a *can1Δ::Ste2pr-spHIS5 lyp1Δ::Ste3pr-LEU2 his3::hisG leu2Δ0 ura3Δ0 tir2Δ*	[69]
CB11F1 (d)	*MAT*a *can1Δ::Ste2pr-spHIS5 lyp1Δ::Ste3pr-LEU2 his3::hisG leu2Δ0 ura3Δ0 tir3Δ*	[69]
CB17H6 (d)	*MAT*a *can1Δ::Ste2pr-spHIS5 lyp1Δ::Ste3pr-LEU2 his3::hisG leu2Δ0 ura3Δ0 tir4Δ*	[69]

(a) unless indicated, strains are in the Σ1278b background; (b) strain was provided by Elizabeth Craig; (c) W303 strains were provided by Joe Horecka; (d) strains came from an ordered deletion collection in the Σ1278b background [69].

**Table 2:** qPCR primer sets used in this study.

Target	Primers	Reference
*ACT1*	5′-GGCTTCTTTGACTACCTTCCAACA-3′	[70]
	5′-GATGGACCACTTTCGTCGTATTC-3′	
*FLO10*	5′-CCAGTGAAGCGTGGCGTTAAAC-3′	This study
	5′-ACGTTGGTGGGTGTTGTGTAG-3′	
*TIP1*	5′-CTGGCAACAGTGGATTCCAAATTC-3′	This study
	5′-GCAATTTAACAATTGTCTTAG-3′	
*PRY2*	5′-CATCAGCTCCTATTGTGGTTGCT-3′	This study
	5′-GGTTGCAGTTGCTGTAGAAAATG-3′	
*TIR1*	5′-CTGTTGCTTCCTCCAGTGAAAC-3′	This study
	5′-AGGTAGCCTCACTGGAAGAAG-3′	
*TIR2*	5′-TCATCGCTGCTTTACAAAGCGCGGG-3′	This study
	5′-GAAGCAGAAGAAGAAGCAGCTG-3′	
*TIR3*	5′-GCGCCATCCTCAAGTGAAGTTG-3′	This study
	5′-GGAAGAGCTGACAACTTCAC-3′	
*TIR4*	5′-GAAGATTCACTAGACTTGCTGGG-3′	This study
	5′-CATCCTTAGAACCAATCAAGTTG-3′	
*OCH1*	5′-CGTGATCAATTATCGTTTGCGTTT-3′	This study
	5′-TCCGGTGAATACGAACCAGAC-3′	

**Table 3:** Cell wall proteins that are candidate fMAPK pathway targets identified by MASS SPEC and/or comparative RNAseq analysis.

Protein	MS Sample (a)	Number of Peptides (b)	Score (c)	*dig1*Δ (d)	*ste12*Δ	Cell Wall Protein (e)	Process (f)	Function
Bar1 (h)	WT and *dig1*Δ not *ste12*Δ	1 for WT and 15 for *dig1*Δ	8.87 for WT and 145.76 for *dig1* Δ	4.83	0.13	[89]	Protein Processing	Aspartyl protease
Flo10 (h)	*dig1*Δ only	10	110.11	2.08	0.20	[95]	Cell Wall Organization	mucin-like protein with similarity to Flo1p, involved in flocculation
Svs1	*dig1*Δ only	3	88.56	3.00	0.32	[96]	Unknown	Cell wall and vacuoal protein
Gas3	*dig1*Δ only	7	61.12	0.65	0.82	[97]	Unknown	Putative 1,3-beta-glucanosyltransferase, GPI-containing protein
Pir1 (h)	*dig1*Δ only	5	53.73	1.18	1.00	[98]	Cell Wall Organization	O-glycosylated protein required for cell wall stability
Tir4	*dig1*Δ only	5	52.49	2.66	3.54	[99]	Unknown	Cell wall mannoprotein
Sed1 (h)	*dig1*Δ only	3	47.4	0.85	0.95	[100]	Cell Wall Organization	Major stress-induced structural GPI-cell wall glycoprotein
Scw4 (h)	*dig1*Δ only	7	43.21	0.85	0.57	[101]	Cell Wall Organization	Cell wall protein with similarity to glucanases
Ccw12 (h)	*dig1*Δ only	1	39.17	1.08	0.86	[98]	Cell Wall Organization	Cell wall mannoprotein
Fig2	*dig1*Δ only	8	38.58	1.55	1.03	[90]	Morphogenesis; filamentous growth	Cell wall adhesin, mating
Flo11 (h)	*dig1*Δ only	2	31.81	18.68	0.46	[42]	Cell Wall Organization	GPI-anchored cell surface flocculin, pseudohyphal formation
Hpf1	*dig1*Δ only	2	17.6	1.37	1.52	[102]	Cell Wall Organization	Haze-protective mannoprotein
Dan4	*dig1*Δ only	5	13.66	1.45	1.32	[103]	Unknown	Cell wall mannoprotein
Yps7	*dig1*Δ only	1	3.08	0.84	1.08	[104]	Cell Wall Organization	Aspartyl protease
Yar066W	*dig1*Δ only	1	2.26	5.69	0.55	[105]	Unknown	Putative GPI protein
Tip1 (g)	n/a	n/a	n/a	1.32	0.57	[106]	Cell Wall Organization	Major cell wall mannoprotein with possible lipase activity
Pry2 (g)(h)	n/a	n/a	n/a	1.64	0.28	[107]	Cell Wall Organization	Unknown
Och1 (g)	n/a	n/a	n/a	1.30	0.70	[108]	Protein N-linked glycosylation	Mannosyltransferase of the cis-Golgi apparatus

a. Cell wall proteins were identified in wild type (WT) (PC538), *ste12*Δ (PC1079), and the *dig1*Δ (PC3039) mutant by MASS SPEC analysis. Proteins exclusively present in only a subset of samples are shown in the table. b. If the protein of interest was identified by mass spec, then the number of distinct peptides identified in the protein group is reported. Otherwise marked n/a. c. Score is a reflection of confidence in the peptide identified, based on the sum of ion scores of all peptides identified. d. The reported fold change in expression in a *dig1*Δ and *ste12Δ* mutant compared to wild type in a comparative RNAseq experiment; GEO: GSE115657. e. Reference characterizing the protein as a cell wall component. f. Process and function determined by information gathered at SGD (http://www.yeastgenome.org). g. Proteins were identified by comparative expression profiling not based on the MASS SPEC analysis. h. Identified in [26].

## Supplementary Materials

The following are available online at http://www.mdpi.com/2309-608X/4/3/93/s1. Table S1: Cell wall enriched proteins from wild-type cells identified by MASS SPEC analysis; Table S2: Cell wall enriched proteins from *ste12*Δ cells identified by MASS SPEC analysis; Table S3: Cell wall enriched proteins from *dig1*Δ cells identified by MASS SPEC analysis; Table S4: Control for MASS SPEC analysis.
